# Intoxicações por cosméticos: análise de série temporal, Brasil, 2019-2025

**DOI:** 10.1590/S2237-96222026v35e20260206.pt

**Published:** 2026-11-09

**Authors:** Daniel Marques Mota, Nelio Cézar de Aquino, Leonardo Oliveira Leitão, Marcus Tolentino Silva

**Affiliations:** 1 Agência Nacional de Vigilância Sanitária, Brasília, DF, Brasil; 2 Universidade de Brasília, Brasília, DF, Brasil

**Keywords:** Centros de Controle de Intoxicações, Cosméticos, Exposição a Produtos Químicos, Vigilância de Produtos Comercializados, Estudos de Séries Temporais, Centros de Control de Intoxicaciones, Cosméticos, Exposición a Compuestos Químicos, Vigilancia de Productos Comercializados, Estudios de Series Temporales

## Abstract

**Objetivo:**

Descrever o perfil epidemiológico e a tendência temporal das intoxicações por cosméticos no Brasil entre 2019 e 2025. Estudo de série temporal com base em dados secundários do Sistema Brasileiro de Dados de Intoxicações. Foram incluídos todos os casos de intoxicação humana por cosméticos. Calcularam-se taxas de incidência anuais e específicas por faixa etária e sexo, razões de taxas e intervalos de confiança de 95% (IC95%). A tendência temporal das taxas anuais foi avaliada por regressão linear simples, em análise exploratória.

**Resultados:**

Foram identificados 14.248 casos de intoxicação por cosméticos. A taxa de incidência aumentou de 0,51 (IC95% 0,48; 0,54) para 1,62 (IC95% 1,57; 1,68) por 100 mil habitantes entre 2019 e 2025. Crianças de 0 a 4 anos representaram 73,7% dos casos e apresentaram razão de taxas de 11,4 (IC95% 11,11; 11,69) em relação à taxa geral do período (6,7; IC95% 6,56; 6,78). Exposições acidentais corresponderam a 85,0% dos registros, e por via oral a 81,7%. Casos leves representaram 59,6% das intoxicações, enquanto casos graves corresponderam a 0,3%, e fatais a menos de 0,1%. Os produtos mais envolvidos foram cosméticos para cabelos e couro cabeludo (21,3%), produtos para unhas (18,5%) e perfumes (13,1%).

**Conclusão:**

As intoxicações por cosméticos aumentaram no Brasil entre 2019 e 2025 e afetaram principalmente crianças pequenas, em exposições acidentais e por via oral, com predominância de casos leves.

## RESUMEN

**Objetivo:**

Describir el perfil epidemiológico y la tendencia temporal de las intoxicaciones por cosméticos en Brasil entre 2019 y 2025.

**Métodos:**

Estudio de serie temporal basado en datos secundarios del Sistema Brasileño de Datos de Intoxicaciones (*Sistema Brasileiro de Dados de Intoxicações*, Datatox). Se incluyeron todos los casos de intoxicación humana por cosméticos. Se calcularon tasas de incidencia anuales y específicas por grupo de edad y sexo, razones de tasas e intervalos de confianza del 95% (IC95%). La tendencia temporal de las tasas anuales se evaluó mediante una regresión lineal simple en un análisis exploratorio.

**Resultados:**

Se identificaron 14.248 casos de intoxicación por cosméticos. La tasa de incidencia aumentó de 0,51 (IC95% 0,48; 0,54) a 1,62 (IC95% 1,57; 1,68) por 100.000 habitantes entre 2019 y 2025. Los niños de 0 a 4 años representaron el 73,7% de los casos y presentaron una razón de tasas de 11,4 (IC95% 11,11; 11,69) en relación con la tasa general del período (6,7; IC95% 6,56; 6,78). Las exposiciones accidentales representaron el 85,0% de los registros y la vía oral, el 81,7%. Los casos leves representaron el 59,6% de las intoxicaciones, mientras que los casos graves representaron el 0,3% y los fatales, menos del 0,1%. Los productos más involucrados fueron: cosméticos para el cabello y el cuero cabelludo (21,3%), productos para las uñas (18,5%) y perfumes (13,1%).

**Conclusión:**

Las intoxicaciones por cosméticos aumentaron en Brasil entre 2019 y 2025 y afectaron principalmente a niños pequeños, debido a exposiciones accidentales y a la vía oral, con predominio de casos leves.

## Introdução

Os cosméticos, abrangendo produtos de higiene pessoal e perfumes, são amplamente utilizados e, geralmente, associados ao menor risco à saúde quando comparados a medicamentos e produtos de limpeza doméstica [1]. Entretanto, a dinâmica atual do mercado de cosmético, caracterizada por intensa inovação, ampla disponibilidade e diversidade de produtos, crescente complexidade das formulações e mudanças no perfil de consumo, incluindo exposição precoce, tem contribuído para um cenário emergente de eventos adversos relacionados a cosméticos, o que inclui as intoxicações [1-4]. 

As intoxicações por cosméticos diferem de outros tipos de exposição química quanto à toxicidade intrínseca, às circunstâncias de ocorrência e ao perfil das populações acometidas. Em geral, envolvem produtos destinados ao uso tópico, com formulações variadas e ampla disponibilidade no ambiente domiciliar. Crianças pequenas constituem o principal grupo afetado por exposições acidentais relacionadas a cosméticos, frequentemente por ingestão oral [1,2]. Diferenças nos sistemas de vigilância, nos contextos regulatórios e nos padrões de uso limitam a extrapolação direta desses achados para o cenário brasileiro, o que reforça a necessidade de análises com base em dados nacionais.

No Brasil, embora a cosmetovigilância seja reconhecida como componente essencial da vigilância pós-comercialização [5], há número limitado de publicações epidemiológicas sobre intoxicações relacionadas a cosméticos. A ocorrência de eventos adversos associados ao uso de cosméticos capilares motivou a adoção de medidas regulatórias e ações de mitigação de risco, o que ressaltou a importância da vigilância pós-comercialização para identificar e gerenciar problemas de segurança relacionados a esses produtos [6,7]. Essa lacuna dificulta tanto a compreensão da magnitude real do problema quanto o planejamento de estratégias preventivas no âmbito da cosmetovigilância. Esse cenário é relevante, pois o mercado brasileiro de cosméticos ocupou a terceira posição global em 2024 [8], o que demanda sistema de vigilância proporcional ao seu impacto na saúde dos consumidores.

O monitoramento das intoxicações por cosméticos no Brasil também se insere no contexto da vigilância toxicológica. O Sistema Brasileiro de Dados de Intoxicações (Datatox), operado pela Associação Brasileira de Centros de Informação e Assistência Toxicológica (Abracit) e alimentado pela rede nacional de Centros de Informação e Assistência Toxicológica (CIATox), representa importante base de dados toxicológicos do país [9,10]. Contudo, o potencial desse sistema permanece subaproveitado na produção científica e na fundamentação de ações regulatórias. 

Utilizar dados coletados por sistemas de vigilância toxicológica permite descrever padrões epidemiológicos e identificar grupos populacionais mais expostos, subsidiando ações de prevenção e estratégias de cosmetovigilância. A análise de séries temporais contribui para avaliar tendências ao longo do tempo, considerando às limitações inerentes aos sistemas de notificação passiva. Este estudo teve como objetivo descrever o perfil epidemiológico e a tendência temporal das intoxicações por cosméticos no Brasil entre 2019 e 2025.

## Métodos

### Desenho do estudo

Trata-se de estudo de série temporal com base em dados secundários de intoxicações por cosméticos do Datatox entre 2019 e 2025. 

### Contexto

O Datatox é um sistema nacional de vigilância toxicológica de caráter passivo, mantido pela Abracit, que integra informações provenientes de 32 CIATox distribuídos nas cinco regiões do Brasil [11]. O sistema consolida dados de atendimentos toxicológicos realizados por telefone, de forma presencial e busca ativa, e constitui fonte nacional de vigilância toxicológica [10]. Para fins deste estudo, o termo “cosmético(s)” abrange produtos de higiene pessoal e perfumes [5].

### Participantes

Foram incluídos todos os casos humanos de intoxicação registrados no Datatox no período do estudo, nos quais cosméticos e produtos de higiene pessoal foram identificados como agente isolado ou em associação com outros agentes tóxicos. 

Não foram aplicados critérios de exclusão. Para selecionar os casos, foram utilizados os filtros “Tipo de vítima=humana” e “Grupo de agentes=cosméticos e higiene pessoal” na interface pública do Datatox [10]. 

Registros com informações incompletas foram mantidos na base analítica e excluídos apenas dos denominadores específicos das variáveis não informadas, uma vez que os dados são previamente consolidados e validados pela rede de CIATox.

### Variáveis

As variáveis temporais incluíram: ano (2019-2025); mês de ocorrência da intoxicação, sendo a distribuição mensal agregada por trimestre; dia da semana, categorizado em dias úteis e finais de semana; e turno do atendimento (madrugada: 0h00min-5h59min; manhã: 6h00min-11h59min; tarde: 12h00min-17h59min; noite: 18h00min-23h59min). 

As variáveis geográficas corresponderam à região geográfica (Norte, Nordeste, Centro-Oeste, Sudeste, Sul) e à Unidade da Federação (UF) do solicitante. O Datatox registra a UF de origem da solicitação, não sendo possível identificar sistematicamente o local de ocorrência da intoxicação ou o município de residência da vítima. 

As variáveis demográficas analisadas incluíram sexo (masculino, feminino, ignorado) e faixa etária, estratificada em grupos padronizados (0-4, 5-9, 10-14, 15-19, 20-29, 30-39, 40-49, 50-59, 60 anos ou mais). 

As características da exposição compreenderam circunstância da exposição e via de exposição. Para fins analíticos, as categorias de circunstância da exposição foram agrupadas em acidental, tentativa de suicídio, reação adversa e outras. Registros classificados como “não se aplica” e “ignorado” foram excluídos das análises. Categorias de menor frequência, incluindo erro de medicação, uso indevido, abuso, ocupacional e tentativa de abortamento, foram agrupadas na categoria “outras”.

As vias de exposição foram agrupadas em oral, cutânea, associação de vias, ocular, respiratória/inalatória e outras. Para essa variável, registros classificados como “não se aplica” e “ignorado” também foram excluídos das análises. Categorias de menor frequência, incluindo exposição nasal e parenteral, foram agrupadas na categoria “outras”. O sistema permitiu o registro de múltiplas circunstâncias e/ou vias de exposição por solicitação, motivo pelo qual os totais dessas variáveis podem exceder o número de casos analisados.

O local de origem dos casos foi categorizado em serviços de saúde (hospitais, unidades de pronto atendimento e outros serviços de saúde - esses últimos incluíram unidades básicas de saúde, centros/postos de saúde, serviços de toxicologia, consultórios/clínicas, farmácias e serviços móveis de atendimento pré-hospitalar), residência e outros locais (aplicativos de mensagem, local de trabalho, escola/creche etc.).

O agente tóxico envolvido foi classificado segundo categorias padronizadas de produtos cosméticos disponíveis no Datatox para fins de registro toxicológico, o que incluiu cosméticos para cabelos e couro cabeludo, produtos para unhas e cutículas, perfumes, repelentes e produtos não especificados. Essas categorias apresentaram correspondência parcial com classes regulatórias utilizadas pela Anvisa para produtos cosméticos [12]. Admitiu-se a possibilidade de mais de um produto cosmético por caso, o que pode resultar em número de registros superior ao total de casos analisados.

As variáveis relativas à gravidade e aos desfechos incluíram a classificação de gravidade (leve, nula, moderada, grave, fatal) e o desfecho (cura, assintomático, cura provável, outros desfechos, sequela, óbito).

### Fontes de dados e mensuração

Os dados foram extraídos em planilhas eletrônicas em 1º de janeiro de 2026 (7h23min) do painel público do Datatox [10], em formato agregado e anonimizado. 

Os dados foram analisados conforme disponibilizados, sem acesso a identificadores individuais. Não foi realizada vinculação individual de bases de dados. A relação entre casos e solicitações foi preservada conforme o registro original do sistema, reconhecendo-se que um mesmo caso pode originar mais de uma solicitação ao longo do seguimento clínico. 

O Datatox admite múltiplos registros associados a um mesmo caso ao longo do acompanhamento toxicológico, de modo que algumas variáveis podem apresentar totais diferentes do número consolidado de casos. Não foi realizada deduplicação adicional além daquela implementada pelo próprio Datatox.

O banco de dados agregado utilizado nas análises foi depositado no repositório SciELO Data [13].

### Potenciais fontes de viés

Este estudo está sujeito a limitações inerentes ao uso de dados de vigilância toxicológica passiva, que inclui: i) subnotificação, particularmente em regiões com menor cobertura da rede CIATox e para casos de intoxicações de menor gravidade; ii) viés de informação, decorrente do registro da UF do solicitante, em vez do local de ocorrência da intoxicação; e iii) viés temporal, relacionado a possíveis mudanças na cobertura e na sensibilidade do Datatox ao longo do período analisado. A utilização de dados agregados impossibilitou o controle de possíveis fatores de confusão individuais.

### Tamanho do estudo

O tamanho do estudo correspondeu ao total de casos humanos de intoxicação por cosméticos registrados no Datatox entre 2019 e 2025.

### Métodos estatísticos

Realizou-se análise estatística descritiva das variáveis. As variáveis categóricas foram expressas em frequências absolutas e relativas, excluindo-se categorias não informativas (“não se aplica” ou “ignorado”) apenas dos denominadores específicos. 

Taxas de incidência anuais foram calculadas por meio de estimativas populacionais do Departamento de Informática do Sistema Único de Saúde (DataSUS) [14], expressas com intervalos de confiança de 95% (IC95%), assumindo distribuição de Poisson. Também foram calculadas taxas específicas por faixa etária e sexo.

Razões de taxas (RTs) foram empregadas como medida exclusivamente descritiva de comparação interna, tendo como referência a taxa geral do período para comparações entre faixas etárias e o sexo masculino para comparação entre sexos. Os IC95% das RTs foram estimados pelo método log-normal.

A variação percentual entre 2019 e 2025 foi calculada como: [(valor2025-valor2019)/valor2019]×100. A taxa de crescimento anual composta (CAGR) foi estimada pela fórmula: CAGR=[(valor final/valor inicial)^(1/n)-1]×100, em que n corresponde ao número de anos do intervalo analisado (n=6). A taxa de crescimento anual composta foi calculada separadamente para casos e taxas de incidência.

A variação temporal das taxas anuais de incidência foi explorada por regressão linear simples, considerando o reduzido número de pontos anuais da série. A taxa de incidência anual foi utilizada como variável dependente e o ano calendário como variável independente. Os resultados dessa análise foram interpretados como descrição exploratória da variação temporal, sem modelagem de autocorrelação ou de pontos de inflexão. A qualidade do ajuste do modelo foi avaliada pelo coeficiente de determinação (R²). Intervalos de confiança de 95% foram estimados com base na distribuição t-Student. Adotou-se nível de significância de 5% (p<0,05).

A densidade de cobertura dos CIATox foi calculada como número de centros por 10 milhões de habitantes, utilizando as estimativas populacionais do DataSUS referidas anteriormente. Os dados da rede CIATox foram obtidos no portal da Abracit, referente à configuração de 2025 [11].

As análises descritivas, os cálculos das taxas de incidência geral e específicas, o IC95%, a RT, a variação percentual e a taxa de crescimento anual composta, bem como a elaboração da representação gráfica da série temporal e da linha de tendência linear, foram realizadas em planilha eletrônica (Microsoft Excel, versão 2021). A análise de regressão linear simples foi conduzida no software Jamovi (versão 2.7.5, The Jamovi Project, 2024).

## Resultados

No conjunto das intoxicações notificadas entre 2019 e 2025 (n=882.092), os cosméticos (isolado+associação) representaram 1,6% dos registros, ocupando a 11ª posição entre agentes tóxicos. Excluindo-se intoxicações por animais peçonhentos/venenosos (n=269.434), animais não peçonhentos (n=30.583) e plantas/fungos (n=11.014), cosméticos corresponderam a 2,5% das 571.061 intoxicações por produtos químicos, posicionando-se atrás de medicamentos (49,7%), produtos domissanitários (8,9%), produtos químicos residenciais/industriais (6,8%) e agrotóxicos (4,8%).

Foram identificados 14.248 casos de intoxicação por cosméticos, com média de 2.035 casos por ano. Desse total, 13.485 (94,6%) envolveram cosméticos como agente isolado, e 763 (5,4%) ocorreram em associação com outros agentes tóxicos. O atendimento telefônico respondeu por 91,0% dos casos, enquanto atendimentos presenciais corresponderam a 1,6%.

O número de casos cresceu 228,9% entre 2019 (n=1.052) e 2025 (n=3.460), com taxa de crescimento anual composta de 21,9% ao ano. A taxa de incidência anual aumentou de 0,51 para 1,62 por 100 mil habitantes, com incremento de 217,6% e taxa de crescimento anual composta de 21,4% ao ano (Tabela 1).

**Tabela 1:** Número e proporção de casos, taxas de incidência por 100 mil habitantes (TI) e intervalo de confiança de 95% (IC95%) e variação anual (%) das intoxicações por cosméticos. Brasil, 2019-2025 (n=14.248)

Ano	n (%)^a^	População	TI/100 mil habitantes (IC95%)	Variação (%)^b^
2019	1.052 (7,4)	207.900.099	0,51 (0,48; 0,54)	-
2020	1.191 (8,4)	209.164.889	0,57 (0,54; 0,60)	+13,2
2021	1.370 (9,6)	210.103.642	0,65 (0,62; 0,69)	+15,0
2022	1.984 (13,9)	210.862.983	0,94 (0,90; 0,98)	+44,8
2023	2.453 (17,2)	211.695.158	1,16 (1,11; 1,20)	+23,6
2024	2.738 (19,2)	212.583.750	1,29 (1,24; 1,34)	+11,6
2025	3.460 (24,3)	213.421.037	1,62 (1,57; 1,68)	+26,4

a Percentual em relação ao total de casos do período analisado; ^b^Variação percentual em relação ao total de casos do ano anterior.

Em 2022, o crescimento anual foi 44,8% em relação a 2021, e em 2025 foi de 26,4%. Na análise exploratória por regressão linear simples, observou-se aumento médio anual de 0,189 casos por 100 mil habitantes (IC95% 0,14; 0,23; p-valor<0,001), entre 2019 e 2025 (Figura 1). A análise da distribuição mensal evidenciou que o último trimestre concentrou 28,1% dos casos; dezembro registrou 1.374 casos. Os atendimentos concentraram-se em dias úteis (73,8%) e no período noturno (40,6%).

As regiões Sudeste e Sul concentraram 77,0% das solicitações com informação válida da UF, enquanto o Norte respondeu por 2,8% (Tabela 2). Entre as UFs, São Paulo concentrou 38,0% das solicitações, seguido por Santa Catarina (11,9%) e Espírito Santo (10,0%). Solicitações sem informação da UF corresponderam a 2,5% (n=472). No total, foram registradas 18.563 solicitações associadas a 14.248 casos, o que resultou na razão de 1,3 solicitação por caso. A produtividade regional dos CIATox variou de 252 solicitações por centro na região Norte a 980 solicitações por centro na região Sudeste.

**Figura 1:**
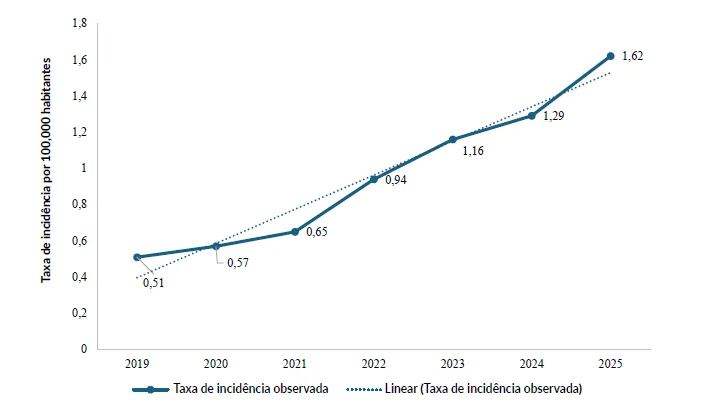
Tendência temporal das taxas de incidência por 100 mil habitantes de intoxicações por cosméticos. Brasil, 2019-2025

Nota(s): A linha contínua com marcadores representa as taxas anuais observadas por 100 mil habitantes. A linha tracejada indica a tendência estimada por regressão linear simples.

**Tabela 2:** Número e proporção de Centros de Informação e Assistência Toxicológica (CIATox) e de solicitações, segundo região geográfica. Brasil, 2019-2025

Região	CIATox, n (%)	População^a^	Densidade^a^	Solicitações, n (%)^b^
Sudeste	10 (31,3)	88.825.643	1,13	9.796 (54,1)
Sul	8 (25,0)	31.310.809	2,56	4.135 (22,9)
Nordeste	8 (25,0)	57.244.485	1,40	2.396 (13,2)
Centro-Oeste	4 (12,5)	17.238.818	2,32	1.259 (7,0)
Norte	2 (6,3)	18.801.282	1,06	505 (2,8)
Total	32 (100,0)	213.421.037	1,50	18.091 (100,0)

a População média do período analisado e densidade de CIATox por 10 milhões de habitantes; ^b^Solicitações acumuladas no período 2019-2025, segundo por região geográfica do solicitante, excluindo-se 472 registros sem informação da Unidade da Federação.

As análises de faixa etária foram realizadas com base em 14.141 registros válidos, excluindo-se registros classificados como “não se aplica”, “ignorado” ou com faixa etária não informada (Tabela 3). Crianças de 0-4 anos concentraram 73,7% (n=10.429) dos casos analisados. Crianças menores de 1 ano corresponderam a 13,0% (n=1.838), enquanto a faixa etária 1-4 anos reuniu 60,7% (n=8.591) das intoxicações. A taxa de incidência específica em crianças de 0-4 anos foi de 76,1 por 100 mil habitantes (IC95% 74,62; 77,54). A faixa etária 5-9 anos apresentou taxa de incidência de 5,2 por 100 mil habitantes (IC95% 4,88; 5,62) (Tabela 3). Em relação à taxa geral do período (6,7 por 100 mil habitantes), crianças de 0-4 anos apresentaram RT de 11,4 (IC95% 11,11; 11,69). Casos sem informação válida de idade corresponderam a 4,0% dos registros excluídos da análise (n=579).

O sexo feminino respondeu por 53,3% dos casos, enquanto o masculino respondeu por 46,7% dos dados informados (n=14.185). As taxas de incidência alcançaram 7,0 por 100 mil habitantes (IC95% 6,84; 7,16) entre mulheres e 6,4 por 100 mil habitantes (IC95% 6,29; 6,60) entre homens, o que resultou na RT de 1,09 (IC95% 1,05; 1,12). 

A análise por faixa etária revelou distribuição equilibrada entre os sexos em menores de 15 anos (51,0% feminino [F] versus 49,0% masculino [M]), com predomínio do sexo feminino a partir dos 15 anos (70,8% dos casos; razão F:M de 2,4:1). A faixa etária 50-59 anos apresentou 75,8% de casos femininos (razão F:M de 3,1:1). Foram excluídos 535 registros sem informação para a variável sexo.

**Tabela 3:** Número e proporção de casos válidos e taxas de incidência por 100 mil habitantes (TI) e razões de taxas (RTs) com intervalos de confiança de 95% (IC95%) das intoxicações por cosméticos, segundo faixa etária. Brasil, 2019-2025 (n=14.141)

Faixa etária (anos)	n (%)^a^	População^b^	TI por 100 mil habitantes (IC95%)	RT (IC95%)^c^
0-4	10.429 (73,7)	13.708.193	76,1 (74,62; 77,54)	11,4 (11,11; 11,69)
5-9	763 (5,4)	14.538.190	5,2 (4,88; 5,62)	0,79 (0,73; 0,85)
10-14	369 (2,6)	14.675.808	2,5 (2,26; 2,77)	0,38 (0,34; 0,42)
15-19	379 (2,7)	15.432.098	2,5 (2,21; 2,70)	0,37 (0,33; 0,41)
20-29	655 (4,6)	32.723.137	2,0 (1,85; 2,15)	0,30 (0,28; 0,32)
30-39	509 (3,6)	33.080.129	1,5 (1,41; 1,67)	0,23 (0,21; 0,25)
40-49	432 (3,1)	30.287.769	1,4 (1,29; 1,56)	0,21 (0,19; 0,24)
50-59	293 (2,1)	24.342.065	1,2 (1,07; 1,34)	0,18 (0,16; 0,20)
≥60	312 (2,2)	33.080.129	0,9 (0,84; 1,05)	0,14 (0,13; 0,16)
Total	14.141 (100,0)	211.867.518	6,7 (6,56; 6,78)	-

a Excluídos registros classificados como “não se aplica” (n=472), “ignorado” (n=27) e faixas etárias ignoradas (n=80); ^b^População média de 2019-2025; ^c^RTs: razão entre a taxa específica por faixa etária e a taxa geral do período (6,7 por 100 mil habitantes).

**Tabela 4:** Características das intoxicações por cosméticos, segundo circunstância e via de exposição, local de origem dos casos, classificação de gravidade e desfecho, com base em registros válidos. Brasil, 2019-2025

Característica	Categoria	n (%)
Circunstância da exposição (n=14.190)	Acidental	12.057 (85,0)
	Tentativa de suicídio	765 (5,4)
	Reação adversa	534 (3,7)
	Outras (uso indevido, abuso, ocupacional etc.)	834 (5,9)
Via de exposição (n=14.229)	Oral	11.625 (81,7)
	Cutânea	897 (6,3)
	Associação de vias	732 (5,1)
	Ocular	408 (2,9)
	Respiratória/inalatória	316 (2,2)
	Outras	251 (1,8)
Local de origem dos casos (n=14.248)	Serviços de saúde	8.644 (60,7)
	Hospitais	4.722 (33,1)
	Unidades de pronto atendimento	2.228 (15,6)
	Outros serviços de saúde	1.694 (11,9)
	Residência	5.208 (36,5)
	Outros (aplicativo de mensagem, local de trabalho, escola/creche etc.)	396 (2,8)
Classificação de gravidade (n=13.107)	Leve	7.802 (59,6)
	Nula	4.915 (37,5)
	Moderada	348 (2,7)
	Grave	34 (0,3)
	Fatal	8 (<0,1)
Desfecho (n=12.138)	Cura	4.701 (38,7)
	Assintomático	4.452 (36,7)
	Cura provável	2.762 (22,8)
	Outros desfechos	213 (1,8)
	Sequela	4 (<0,1)
	Óbito	6 (<0,1)

Entre os registros válidos de circunstância da exposição (n=14.190), exposições acidentais corresponderam a 85,0% (Tabela 4). Entre os registros válidos de via de exposição (n=14.229), predominou a via oral (81,7%). Serviços de saúde representaram a origem de 60,7% dos casos (n=14.248), enquanto casos leves corresponderam a 59,6% das classificações de gravidade válidas (n=13.107). Em relação aos desfechos (n=12.138), predominaram cura (38,7%) e casos assintomáticos (36,7%), enquanto óbitos e sequelas foram raros (<0,1%).

Quanto às classes de cosméticos envolvidos, foram analisados 14.774 registros válidos, admitindo-se mais de um produto por caso. Produtos para cabelos e couro cabeludo corresponderam a 21,3% dos registros, seguidos por produtos para unhas e cutículas (18,5%), perfumes (13,1%) e repelentes (12,8%). Cosméticos, produtos de higiene pessoal e perfumes não especificados representaram 10,2% dos registros. As demais classes apresentaram frequências inferiores a 8,0%.

## Discussão

Este estudo observou aumento das taxas de incidência de intoxicações por cosméticos registradas no Datatox no Brasil entre 2019 e 2025. Houve predomínio de exposições acidentais e por via oral. Crianças menores de 5 anos concentraram a maior parte dos casos e apresentaram taxas de incidência superiores às demais faixas etárias. Apesar da elevada frequência de casos originados de serviços de saúde, predominou o registro de casos leves, com baixa ocorrência de desfechos graves ou fatais. Os produtos mais frequentemente envolvidos foram cosméticos para cabelos e couro cabeludo, unhas e cutículas e perfumes.

Este estudo apresentou limitações inerentes ao uso de dados de vigilância passiva. O Datatox capta apenas os casos notificados, o que representa provavelmente uma fração das intoxicações ocorridas no país, uma vez que seus registros dependem de solicitações espontâneas aos CIATox, estando sujeitos à subnotificação. A utilização da UF do solicitante, em vez do local de ocorrência da intoxicação, limita interpretações geográficas mais precisas. A distribuição e a organização dos CIATox no território nacional podem influenciar a representatividade regional dos dados. A classificação dos agentes tóxicos não permite identificar produtos, marcas ou ingredientes específicos, restringindo análises regulatórias mais detalhadas. A variabilidade na completude das informações constitui limitação adicional, aspecto reconhecido em estudos que utilizam bases de dados toxicológicos secundários [15].

O delineamento descritivo do estudo impediu inferências causais. O uso da população geral como denominador pode subestimar o risco real entre consumidores efetivos de cosméticos. Diferenças regionais observadas podem refletir tanto variações na ocorrência das intoxicações quanto desigualdades no acesso aos serviços de toxicologia e na cultura de notificação. A análise temporal adotada possui caráter exploratório, considerando o reduzido número de observações anuais analisadas. A impossibilidade de análise de sensibilidade restrita aos CIATox com funcionamento contínuo ao longo de todo o período limita a distinção entre expansão da rede de vigilância e aumento real na incidência. Estudos futuros com acesso a dados desagregados por centro poderão contribuir para esclarecer essa questão.

O crescimento das taxas de incidência sugere aumento progressivo das intoxicações por cosméticos no país ao longo do período analisado. Embora parte desse crescimento possa estar relacionada à ampliação da cobertura e da capacidade de detecção da rede CIATox, a magnitude do aumento observado foi superior ao crescimento populacional brasileiro no mesmo período. Esse padrão é compatível com aumento da exposição populacional a cosméticos, em contexto de expansão e diversificação do mercado brasileiro desses produtos [8]. Tendência semelhante de crescimento das exposições relacionadas a cosméticos também foi encontrada nos Estados Unidos em 2023 [1].

O aumento das intoxicações observado no período provavelmente resulta de múltiplos fatores relacionados ao maior acesso e à disponibilidade de cosméticos no ambiente domiciliar. Houve aumento de exposições domésticas a produtos de uso cotidiano, o que inclui produtos químicos e de higiene pessoal, especialmente em contextos de maior permanência domiciliar durante e após a pandemia de covid-19 [16,17]. Embora o delineamento deste estudo não permita estabelecer relações causais, mudanças nos padrões de consumo, armazenamento e acesso domiciliar a cosméticos podem ter contribuído parcialmente para as tendências observadas.

A elevada concentração de casos em crianças menores de 5 anos foi consistente com dados internacionais. Crianças pequenas foram identificadas como o principal grupo afetado por exposições acidentais relacionadas a cosméticos, frequentemente associadas à ingestão oral [1,2]. O predomínio observado nessa faixa etária é compatível com características do desenvolvimento infantil e com a maior ocorrência de exposições acidentais em ambiente domiciliar [2,18,19].

Características sensoriais dos cosméticos, como fragrâncias, coloração intensa, embalagens atrativas e semelhança visual com alimentos, também são descritas como fatores associados ao risco de exposições acidentais na infância [2,18]. Soma-se a isso o armazenamento inadequado desses produtos no ambiente domiciliar, a supervisão insuficiente de crianças pequenas e a ausência de exigência regulatória ampla de embalagens resistentes à abertura por crianças para a maioria dos cosméticos comercializados [2,19]. Embora este estudo não permita avaliar diretamente estratégias de marketing, comportamento de consumo ou condições específicas de armazenamento domiciliar, a ampla disponibilidade desses produtos no ambiente doméstico pode contribuir para o padrão epidemiológico observado.

O discreto predomínio do sexo feminino observado no conjunto dos casos deve ser interpretado à luz da distribuição etária das intoxicações. Em menores de 15 anos, especialmente entre crianças pequenas, a distribuição entre os sexos mostrou-se relativamente equilibrada, o que sugere que a exposição está mais relacionada ao ambiente domiciliar e à disponibilidade dos produtos do que a diferenças comportamentais de gênero. A partir da adolescência e nas faixas etárias adultas, observou-se predominância consistente do sexo feminino, com maior frequência entre 50 e 59 anos, padrão compatível com o maior uso e a diversidade de produtos cosméticos por mulheres ao longo da vida adulta descritos na literatura [20].

A razão entre solicitações e casos superior à unidade indica que parte das intoxicações gerou múltiplas consultas aos CIATox, padrão esperado em sistemas de vigilância toxicológica que realizam acompanhamento clínico e orientação a profissionais de saúde e cuidadores [21,22]. As análises epidemiológicas deste estudo foram realizadas com base em casos únicos, o que permitiu estimativas mais adequadas das taxas de incidência e reduziu potencial superestimação da ocorrência das intoxicações.

A elevada frequência de casos originados de serviços de saúde, particularmente hospitais, associada ao predomínio de casos leves e à baixa frequência de desfechos graves ou fatais, sugere que parcela expressiva das exposições motivou avaliação clínica preventiva, especialmente em crianças pequenas. Exposições pediátricas relacionadas a cosméticos e produtos de baixa toxicidade frequentemente motivam busca por atendimento médico e contato com serviços especializados [1,2,23]. A elevada proporção de casos assintomáticos, curas e curas prováveis observada neste estudo reforça esse perfil clínico predominantemente benigno.

As categorias de produtos mais frequentemente envolvidas - cosméticos para cabelos e couro cabeludo, produtos para unhas e cutículas e perfumes - apresentaram similaridade com dados norte-americanos [2]. Esses produtos costumam estar amplamente disponíveis no ambiente domiciliar e apresentam características sensoriais atrativas para crianças pequenas, como fragrâncias, coloração intensa e embalagens acessíveis [2,18,19], o que favorece exposições acidentais.

As intoxicações por cosméticos registradas no Datatox aumentaram no Brasil entre 2019 e 2025 e afetaram predominantemente crianças pequenas em exposições acidentais e por via oral. O padrão epidemiológico caracterizou-se por predomínio de casos leves e baixa frequência de desfechos graves ou fatais. Os achados contribuem para a caracterização epidemiológica dessas intoxicações no país e podem subsidiar ações de vigilância toxicológica, cosmetovigilância e prevenção voltadas a populações mais expostas.

## Data Availability

O banco de dados agregado utilizado neste estudo foi depositado no repositório SciELO Data e está disponível em: https://doi.org/10.48331/SCIELODATA.ZT6PK4. Os dados correspondem à extração realizada do painel público Datatox/Abracit em 1º de janeiro de 2026, às 7h23min.
