# Supplementary material for: Intoxicações por cosméticos: análise de série temporal, Brasil, 2019-2025
Source: Epidemiol Serv Saude. 2026 Nov 9;35:e20260206. [Article in Portuguese] doi: 10.1590/S2237-96222026v35e20260206.pt (PMC13750722; doi:10.1590/S2237-96222026v35e20260206.pt)
Supplement: Translation

# Translation: Cosmetic poisoning: time series analysis, Brazil, 2019-2025

**DOI:** 10.1590/S2237-96222026v35e20260206.en

**Authors:** Daniel Marques Mota, Nelio Cézar de Aquino, Leonardo Oliveira Leitão, Marcus Tolentino Silva

**Affiliations:** 1 Agência Nacional de Vigilância Sanitária, Brasília, DF, Brazil; 2 Universidade de Brasília, Brasília, DF, Brazil

**Keywords:** Poison Control Centers, Cosmetics, Chemical Compound Exposure, Product Surveillance, Postmarketing, Time Series Studies

## Abstract

**Objective:**

To describe the epidemiological profile and temporal trend of cosmetic poisoning in Brazil between 2019 and 2025.

**Methods:**

A time series study based on secondary data from the Brazilian Poisoning Data System (*Sistema Brasileiro de Dados de Intoxicações*, Datatox). All cases of human cosmetic poisoning were included. Annual and age- and sex-specific incidence rates, rate ratios, and 95% confidence intervals (95%CI) were calculated. The temporal trend of annual incidence rates was assessed using simple linear regression in an exploratory analysis.

**Results:**

A total of 14,248 cases of cosmetic poisoning were identified. The incidence rate increased from 0.51 (95%CI 0.48; 0.54) to 1.62 (95%CI 1.57; 1.68) per 100,000 population between 2019 and 2025. Children aged 0-4 years accounted for 73.7% of cases and had a rate ratio of 11.4 (95%CI 11.11; 11.69) compared with the overall incidence rate for the study period (6.7; 95%CI 6.56; 6.78). Accidental exposures accounted for 85.0% of records, and oral exposure for 81.7%. Mild cases accounted for 59.6% of poisonings, whereas severe cases accounted for 0.3%, and fatal cases for less than 0.1%. The products most frequently involved were hair and scalp cosmetics (21.3%), nail products (18.5%), and perfumes (13.1%).

**Conclusion:**

Cosmetic poisoning increased in Brazil between 2019 and 2025 and predominantly affected young children through accidental oral exposures, with a predominance of mild cases.

## Introduction

Cosmetics, including personal care products and perfumes, are widely used and generally associated with lower health risks than medications and household cleaning products [1]. However, the current dynamics of the cosmetics market, characterized by intense innovation, broad product availability and diversity, increasing formulation complexity, and shifts in consumption patterns, including early-life exposure, have contributed to an emerging landscape of cosmetic-related adverse events, including poisonings [1-4]. 

Cosmetic poisoning differs from other types of chemical exposure in terms of intrinsic toxicity, circumstances of occurrence, and the profile of affected populations. In general, these events involve products intended for topical use, with diverse formulations and widespread availability in the household environment. Young children are the primary group affected by accidental exposures to cosmetics, often through ingestion [1,2]. Differences in surveillance systems, regulatory contexts, and patterns of use limit the direct extrapolation of these findings to the Brazilian context, reinforcing the need for analyses based on national data.

In Brazil, although cosmetovigilance is recognized as an essential component of post-marketing surveillance [5], epidemiological publications on cosmetic-related poisoning remain limited. The occurrence of adverse events associated with hair cosmetics prompted regulatory measures and risk mitigation actions, underscoring the importance of post-marketing surveillance for identifying and managing safety issues related to these products [6,7]. This knowledge gap hinders both understanding of the problem’s true magnitude and planning preventive strategies within the scope of cosmetovigilance. This scenario is particularly relevant because the Brazilian cosmetics market ranked third globally in 2024 [8], requiring a surveillance system commensurate with its impact on consumer health.

Monitoring cosmetic poisoning in Brazil is also part of toxicological surveillance. The Brazilian Poisoning Data System (*Sistema Brasileiro de Dados de Intoxicações*, Datatox), operated by the Brazilian Association of Toxicological Information and Assistance Centers (*Associação Brasileira de Centros de Informação e Assistência Toxicológica*, Abracit) and receiving data from the Toxicological Information and Assistance Centers (*Centros de Informação e Assistência Toxicológica*, CIATox) national network, represents an important toxicological database in the country [9,10]. However, the potential of this system remains underutilized in scientific research and in supporting regulatory actions. 

The use of data collected by toxicological surveillance systems enables the description of epidemiological patterns and the identification of population groups at greater risk of exposure, thereby supporting prevention measures and cosmetovigilance strategies. Time series analysis helps assess temporal trends while acknowledging the limitations inherent in passive surveillance systems. This study aimed to describe the epidemiological profile and temporal trend of cosmetic poisoning in Brazil between 2019 and 2025.

## Methods

### Study design

This is a time series study using secondary data on cosmetic poisoning from Datatox between 2019 and 2025. 

### Setting

Datatox is a national passive toxicological surveillance system, maintained by Abracit, which integrates information from 32 CIATox distributed across the five regions of Brazil [11]. The system consolidates data from toxicological consultations conducted by telephone, in person, and through active case finding, and constitutes a national source of toxicological surveillance [10]. For this study, the term “cosmetics” encompasses personal care products and perfumes [5].

### Participants

All human cases of poisoning recorded in Datatox during the study period, in which cosmetics and personal care products were identified as an isolated agent or in association with other toxic agents, were included. 

No exclusion criteria were applied. To select cases, the filters “Type of victim=human” and “Agent group=cosmetics and personal care” were used in the public Datatox interface [10]. 

Records with incomplete information were retained in the analytical database and excluded only from the denominators of variables with missing values, since the data had been previously consolidated and validated by the CIATox network.

### Variables

Temporal variables included: year (2019-2025); month of poisoning occurrence, with monthly distribution aggregated by quarter; day of the week, categorized as weekdays and weekends; and time of attendance (early morning: 00:00-05:59; morning: 06:00-11:59; afternoon: 12:00-17:59; night: 18:00-23:59). 

Geographical variables corresponded to the geographic region (North, Northeast, Central-West, Southeast, South) and to the Federation Unit of the requester. Datatox records the Federation Unit of origin of the request, making it impossible to systematically identify the place of occurrence of the poisoning or the victim’s municipality of residence. 

Demographic variables analyzed included sex (male, female, unknown) and age group, stratified into standardized groups (0-4, 5-9, 10-14, 15-19, 20-29, 30-39, 40-49, 50-59, 60 years or older). 

Exposure characteristics included the circumstances and route of exposure. For analytical purposes, categories of exposure circumstance were grouped into accidental, suicide attempt, adverse reaction, and others. Records classified as “not applicable” and “unknown” were excluded from the analyses. Low-frequency categories, including medication error, misuse, abuse, occupational exposure, and abortion attempt, were grouped into the “other” category.

Routes of exposure were grouped into oral, cutaneous, multiple routes, ocular, respiratory/inhalational, and others. For this variable, records classified as “not applicable” and “unknown” were also excluded from the analyses. Low-frequency categories, including nasal and parenteral exposure, were grouped into the “others” category. The system allowed the recording of multiple circumstances and/or routes of exposure per report; therefore, totals for these variables may exceed the number of cases analyzed.

The origin of cases was categorized as healthcare services (hospitals, emergency care units, and other healthcare services - the latter including primary health care units, health centers/posts, toxicology services, outpatient clinics, pharmacies, and mobile pre-hospital care services), residence, and other locations (messaging applications, workplace, school/daycare, etc.).

The toxic agent involved was classified according to standardized categories of cosmetic products available in Datatox for toxicological recording purposes, including hair and scalp cosmetics, nail and cuticle products, perfumes, repellents, and unspecified products. These categories showed partial correspondence to the regulatory classes used by the Brazilian Health Regulatory Agency (*Agência Nacional de Vigilância Sanitária*, Anvisa) for cosmetic products [12]. The possibility of more than one cosmetic product per case was acknowledged, which may result in several records exceeding the total number of cases analyzed.

Variables related to severity and outcomes included severity classification (mild, none, moderate, severe, fatal) and outcome (recovery, asymptomatic, probable recovery, other outcomes, sequelae, death).

### Data sources and measurement

Data were extracted into electronic spreadsheets on 1 January 2026 (07:23) from the Datatox public dashboard [10], in aggregated and anonymized format. 

Data were analyzed as made available, without access to individual identifiers. No individual-level data linkage was performed. The relationship between cases and reports was preserved in accordance with the system’s original recording, recognizing that a single case may generate multiple reports during clinical follow-up. 

Datatox allows multiple records associated with the same case over the course of toxicological follow-up; therefore, some variables may show totals that differ from the consolidated case count. No additional deduplication beyond what Datatox implemented was performed.

The aggregated database used in the analyses was deposited in the SciELO Data repository [13].

### Potential sources of bias

This study is subject to limitations inherent to the use of passive toxicological surveillance data, including: i) underreporting, particularly in regions with lower CIATox network coverage and for less severe poisoning cases; ii) information bias, resulting from recording the requester’s Federation Unit rather than the place of occurrence of the poisoning; and iii) temporal bias, related to possible changes in Datatox coverage and sensitivity over the study period. The use of aggregated data prevented control for possible individual confounding factors.

### Study size

The study size corresponded to the total number of human cosmetic poisoning cases recorded in Datatox between 2019 and 2025.

### Statistical methods

Descriptive statistical analysis of variables was performed. Categorical variables were expressed as absolute and relative frequencies, excluding non-informative categories (“not applicable” or “unknown”) only from the denominators of specific variables. 

Annual incidence rates were calculated using population estimates from the Brazilian Unified Health System Information Technology Department (*Departamento de Informação e Informática do Sistema Único de Saúde,* DATASUS) [14], with 95% confidence intervals (95%CI) estimated assuming a Poisson distribution. Age- and sex-specific rates were also calculated.

Rate ratios (RR) were used exclusively as a descriptive measure for internal comparisons, with the overall rate for the study period as the reference for comparisons between age groups and the male sex as the reference for comparisons between sexes. The 95%CI for the RR was estimated using the log-normal method.

The percentage change between 2019 and 2025 was calculated as: [(value2025−value2019)/value2019]×100. The compound annual growth rate (CAGR) was estimated using the formula: CAGR=[(final value/initial value)^(1/n)−1]×100, where n corresponds to the number of years in the analyzed interval (n=6). The compound annual growth rate was calculated separately for cases and incidence rates.

The temporal variation in annual incidence rates was examined using simple linear regression, given the limited number of annual observations in the series. The annual incidence rate was used as the dependent variable, and the calendar year as the independent variable. The results of this analysis were interpreted as an exploratory description of temporal variation, without modeling autocorrelation or inflection points. Model fit quality was assessed using the coefficient of determination (R²). 95% confidence intervals were estimated based on Student’s t-distribution. A significance level of 5% (p<0.05) was adopted.

CIATox coverage density was calculated as the number of centers per 10,000,000 inhabitants, using the previously mentioned DATASUS population estimates. Data on the CIATox network were obtained from the Abracit portal, referring to the 2025 configuration [11].

Descriptive analyses, calculations of overall and specific incidence rates, 95%CI, RR, percentage variation, and CAGR, as well as the construction of the time series graphical representation and linear trend line, were performed using a spreadsheet program (Microsoft Excel, version 2021). Simple linear regression analysis was conducted using Jamovi software (version 2.7.5, The Jamovi Project, 2024).

## Results

Among all poisoning cases reported between 2019 and 2025 (n=882,092), cosmetics (alone or in combination with other agents) accounted for 1.6% of records, ranking 11th among toxic agents. After excluding poisonings involving venomous/poisonous animals (n=269,434), non-venomous animals (n=30,583), and plants/fungi (n=11,014), cosmetics accounted for 2.5% of the 571,061 poisonings involving chemical products, ranking behind medications (49.7%), household cleaning products (8.9%), household/industrial chemicals (6.8%), and pesticides (4.8%).

A total of 14,248 cases of cosmetic poisoning were identified, averaging 2,035 cases per year. Of these, 13,485 (94.6%) involved cosmetics as the sole agent, whereas 763 (5.4%) occurred in association with other toxic agents. Telephone consultations accounted for 91.0% of cases, whereas in-person consultations accounted for 1.6%.

The number of cases increased by 228.9% between 2019 (n=1,052) and 2025 (n=3,460), with a compound annual growth rate of 21.9%. The annual incidence rate increased from 0.51 to 1.62 per 100,000 inhabitants, representing a 217.6% increase and a compound annual growth rate of 21.4% (Table 1).

**Table 1:** Number and proportion of cases, incidence rates per 100,000 inhabitants (IR), 95% confidence intervals (95%CI), and annual variation (%) of cosmetic poisonings. Brazil, 2019-2025 (n=14,248)

Year	n (%)^a^	Population	IR per 100,000 population (95%CI)	Change (%)^b^
2019	1,052 (7.4)	207,900,099	0.51 (0.48; 0.54)	-
2020	1,191 (8.4)	209,164,889	0.57 (0.54; 0.60)	+13.2
2021	1,370 (9.6)	210,103,642	0.65 (0.62; 0.69)	+15.0
2022	1,984 (13.9)	210,862,983	0.94 (0.90; 0.98)	+44.8
2023	2,453 (17.2)	211,695,158	1.16 (1.11; 1.20)	+23.6
2024	2,738 (19.2)	212,583,750	1.29 (1.24; 1.34)	+11.6
2025	3,460 (24.3)	213,421,037	1.62 (1.57; 1.68)	+26.4

^a^Percentage relative to the total number of cases in the study period; ^b^Annual change relative to the total number of cases in the previous year.

In 2022, annual growth was 44.8% compared with 2021, and in 2025 it was 26.4%. Exploratory analysis using simple linear regression showed a mean annual increase of 0.189 cases per 100,000 inhabitants (95%CI 0.14; 0.23; p-value<0.001) between 2019 and 2025 (Figure 1). Analysis of the monthly distribution showed that the fourth quarter accounted for 28.1% of cases; December recorded 1,374 cases. Consultations were concentrated on weekdays (73.8%) and at night (40.6%).

The Southeast and South regions accounted for 77.0% of requests with valid Federation Unit information, whereas the North accounted for 2.8% (Table 2). Among the Federation Units, São Paulo accounted for 38.0% of requests, followed by Santa Catarina (11.9%) and Espírito Santo (10.0%). Requests without Federation Unit information accounted for 2.5% (n=472). A total of 18,563 requests were associated with 14,248 cases, resulting in a ratio of 1.3 requests per case. Regional CIATox productivity ranged from 252 requests per center in the North region to 980 in the Southeast.

**Figure 1:** Temporal trend of incidence rates per 100,000 inhabitants of poisonings by cosmetics. Brazil, 2019-2025

Note(s): The solid line with markers represents the observed annual rates per 100,000 inhabitants. The dashed line indicates the trend estimated by simple linear regression.

**Table 2:** Number and proportion of Toxicological Information and Assistance Centers (*Centros de Informação e Assistência Toxicológica*, CIATox) and requests by geographic region. Brazil, 2019-2025

Region	CIATox, n (%)	Population^a^	Density^a^	Requests, n (%)^b^
Southeast	10 (31.3)	88,825,643	1,13	9,796 (54.1)
South	8 (25.0)	31,310,809	2,56	4,135 (22.9)
Northeast	8 (25.0)	57,244,485	1,40	2,396 (13.2)
Central-West	4 (12.5)	17,238,818	2,32	1,259 (7.0)
North	2 (6.3)	18,801,282	1,06	505 (2.8)
Total	32 (100.0)	213,421,037	1,50	18,091 (100.0)

a Average population for the study period and CIATox density per 10,000,000 inhabitants; ^b^Cumulative requests for the 2019-2025 period, by geographic region of the requester, excluding 472 records with missing Federation Unit information.

Age-group analyses were based on 14,141 valid records, excluding records classified as “not applicable,” “unknown,” or with missing age-group information (Table 3). Children aged 0-4 years accounted for 73.7% (n=10,429) of the analyzed cases. Children younger than 1 year accounted for 13.0% (n=1,838), whereas those aged 1-4 years accounted for 60.7% (n=8,591) of poisonings. The age-specific incidence rate among children aged 0-4 years was 76.1 per 100,000 inhabitants (95%CI 74.62; 77.54). Children aged 5-9 years had an incidence rate of 5.2 per 100,000 inhabitants (95%CI 4.88; 5.62) (Table 3). Compared with the overall incidence rate for the study period (6.7 per 100,000 inhabitants), children aged 0-4 years had an RR of 11.4 (95%CI 11.11; 11.69). Cases without valid age information accounted for 4.0% of the records excluded from the analysis (n=579).

Females accounted for 53.3% of cases, whereas males accounted for 46.7% of records with valid information (n=14,185). Incidence rates reached 7.0 per 100,000 inhabitants (95%CI 6.84; 7.16) among females and 6.4 per 100,000 inhabitants (95%CI 6.29; 6.60) among males, resulting in an RR of 1.09 (95%CI 1.05; 1.12). 

Age-group analysis showed a balanced distribution between the sexes among individuals younger than 15 years (51.0% female [F] versus 49.0% male [M]), with female predominance from 15 years of age onward (70.8% of cases; F:M ratio of 2.4:1). The 50-59-year age group presented 75.8% female cases (F:M ratio of 3.1:1). A total of 535 records without information on sex were excluded.

**Table 3:** Number and proportion of valid cases, incidence rates per 100,000 inhabitants (IR), and rate ratios (RR) with 95% confidence intervals (95%CI) of cosmetic poisonings, by age group. Brazil, 2019-2025 (n=14,141)

Age group (years)	n (%)^a^	Population^b^	TI by 100 thousand inhabitants (95%CI)	RR (95%CI)^c^
0-4	10,429 (73.7)	13,708,193	76.1 (74.62; 77.54)	11.4 (11.11; 11.69)
5-9	763 (5.4)	14,538,190	5.2 (4.88; 5.62)	0.79 (0.73; 0.85)
10-14	369 (2.6)	14,675,808	2.5 (2.26; 2.77)	0.38 (0.34; 0.42)
15-19	379 (2.7)	15,432,098	2.5 (2.21; 2.70)	0.37 (0.33; 0.41)
20-29	655 (4.6)	32,723,137	2.0 (1.85; 2.15)	0.30 (0.28; 0.32)
30-39	509 (3.6)	33,080,129	1.5 (1.41; 1.67)	0.23 (0.21; 0.25)
40-49	432 (3.1)	30,287,769	1.4 (1.29; 1.56)	0.21 (0.19; 0.24)
50-59	293 (2.1)	24,342,065	1.2 (1.07; 1.34)	0.18 (0.16; 0.20)
≥60	312 (2.2)	33,080,129	0.9 (0.84; 1.05)	0.14 (0.13; 0.16)
Total	14,141 (100.0)	211,867,518	6.7 (6.56; 6.78)	-

a Records classified as “not applicable” (n=472), “unknown” (n=27), and missing age group information (n=80) were excluded; ^b^Average population for 2019-2025; ^c^RR: ratio between the age-specific rate and the overall rate for the study period (6.7 per 100,000 inhabitants).

**Table 4:** Characteristics of poisonings by cosmetics, according to circumstance and route of exposure, origin of cases, severity classification, and outcome, based on valid records. Brazil, 2019-2025

Characteristics	Category	n (%)
Circumstance of exposure (n=14,190)	Accidental	12,057 (85.0)
	Suicide attempt	765 (5.4)
	Adverse reaction	534 (3.7)
	Others (misuse, abuse, occupational exposure etc.)	834 (5.9)
Route of exposure (n=14,229)	Oral	11,625 (81.7)
	Cutaneous	897 (6.3)
	Mulitple routes	732 (5.1)
	Ocular	408 (2.9)
	Respiratory/inhalational	316 (2.2)
	Other	251 (1.8)
Origin of cases (n=14,248)	Healthcare services	8,644 (60.7)
	Hospitals	4,722 (33.1)
	Emergency Care Centers	2,228 (15.6)
	Other healthcare facilities	1,694 (11.9)
	Residence	5,208 (36.5)
	Others (messaging application, workplace, school/daycare etc.)	396 (2.8)
Severity classification (n=13,107)	Mild	7,802 (59.6)
	None	4,915 (37.5)
	Moderate	348 (2.7)
	Severe	34 (0.3)
	Fatal	8 (<0.1)
Outcome (n=12,138)	Recovery	4,701 (38.7)
	Asymptomatic	4,452 (36.7)
	Probable recovery	2,762 (22.8)
	Other outcomes	213 (1.8)
	Sequelae	4 (<0.1)
	Death	6 (<0.1)

Among records with valid information on the circumstances of exposure (n=14,190), accidental exposures accounted for 85.0% (Table 4). Among records with valid information on the route of exposure (n=14,229), oral exposure predominated (81.7%). Healthcare services accounted for 60.7% of cases (n=14,248), whereas mild cases accounted for 59.6% of valid severity classifications (n=13,107). Regarding outcomes (n=12,138), recovery (38.7%) and asymptomatic cases (36.7%) predominated, whereas deaths and sequelae were rare (<0.1%).

Regarding the classes of cosmetic products involved, 14,774 valid records were analyzed, allowing for multiple products per case. Hair and scalp cosmetics accounted for 21.3% of records, followed by nail and cuticle products (18.5%), perfumes (13.1%), and repellents (12.8%). Unspecified cosmetics, personal care products, and perfumes accounted for 10.2% of records. The remaining classes each accounted for less than 8.0% of records.

## Discussion

This study observed an increase in the incidence of cosmetic poisoning recorded in Datatox in Brazil between 2019 and 2025. Accidental and oral exposures predominated. Children younger than 5 years accounted for most cases and presented higher incidence rates than the other age groups. Despite the high frequency of cases originating from healthcare services, mild cases predominated, with few severe or fatal outcomes. The products most frequently involved were hair and scalp cosmetics, nail and cuticle products, and perfumes.

This study has limitations inherent to the use of passive surveillance data. Datatox records only report cases, which likely represent only a fraction of the poisonings occurring in the country, since its records depend on spontaneous requests to CIATox and are therefore subject to underreporting. The use of the requester’s Federation Unit, rather than the location where the poisoning occurred, limits the precision of geographical interpretations. The distribution and organization of CIATox across the national territory may affect the data’s regional representativeness. The classification of toxic agents does not allow the identification of specific products, brands, or ingredients, limiting more detailed regulatory analyses. Variability in data completeness is an additional limitation, a point recognized in studies using secondary toxicological databases [15].

The descriptive study design precluded causal inferences. Using the general population as the denominator may underestimate the actual risk among cosmetic users. The regional differences observed may reflect both variations in the occurrence of poisonings and inequalities in access to toxicology services and in reporting practices. The temporal analysis was exploratory, given the limited number of annual observations. The inability to perform a sensitivity analysis restricted to CIATox operating continuously throughout the study period limits the ability to distinguish between expansion of the surveillance network and a true increase in incidence. Future studies with access to center-level disaggregated data may help clarify this issue.

The increase in incidence rates suggests a progressive rise in cosmetic poisoning in the country over the study period. Although part of this increase may be related to the expansion of CIATox network coverage and detection capacity, the magnitude of the observed increase exceeded Brazilian population growth over the same period. This pattern is consistent with increased population exposure to cosmetics amid the expansion and diversification of the Brazilian cosmetics market [8]. A similar increasing trend in cosmetic-related exposures was also reported in the United States in 2023 [1].

The increase in poisonings observed during the study period likely resulted from multiple factors related to greater access to and availability of cosmetics in the household environment. There was an increase in household exposure to everyday-use products, including chemicals and personal care products, particularly during and after the COVID-19 pandemic, when people spent more time at home [16,17]. Although the design of this study does not allow causal inferences, changes in patterns of cosmetic consumption, storage, and household access may have partially contributed to the observed trends.

The high concentration of cases among children younger than 5 years was consistent with international data. Young children have been identified as the main group affected by accidental exposures to cosmetics, frequently associated with oral ingestion [1,2]. The predominance observed in this age group is consistent with the characteristics of early childhood development and the higher incidence of accidental exposures in the household environment [2,18,19].

Sensory characteristics of cosmetics, such as fragrances, vivid colors, attractive packaging, and visual resemblance to food, have also been described as factors associated with the risk of accidental exposure during childhood [2,18]. In addition, inadequate household storage of these products, insufficient supervision of young children, and the absence of broad regulatory requirements for child-resistant packaging for most commercially available cosmetics contribute to this risk [2,19]. Although this study does not permit direct assessment of marketing strategies, consumer behavior, or specific household storage conditions, the widespread availability of these products in household settings may contribute to the observed epidemiological pattern.

The slight predominance of females observed among all cases should be interpreted in light of the age distribution of poisonings. Among individuals under 15 years of age, particularly young children, the distribution between the sexes was relatively balanced, suggesting that exposure is more closely related to the household environment and product availability than to gender-related behavioral differences. From adolescence onward and across adult age groups, females consistently predominated, with the highest frequency among individuals aged 50-59 years, a pattern consistent with the greater use and wider variety of cosmetic products by women throughout adulthood, as described in the literature [20].

The ratio of requests to cases greater than one indicates that some poisonings generated multiple consultations with CIATox, an expected pattern in toxicological surveillance systems that provide clinical follow-up and guidance to healthcare professionals and caregivers [21,22]. The epidemiological analyses in this study were based on unique cases, allowing more appropriate estimates of incidence rates and reducing potential overestimation of poisoning occurrence.

The high frequency of cases originating from healthcare services, particularly hospitals, together with the predominance of mild cases and the low frequency of severe or fatal outcomes, suggests that a substantial proportion of exposures prompted precautionary clinical evaluation, especially among young children. Pediatric exposures involving cosmetics and other low-toxicity products frequently lead to medical evaluation and contact with specialized services [1,2,23]. The high proportion of asymptomatic cases, recoveries, and probable recoveries observed in this study reinforces this predominantly benign clinical profile.

The product categories most frequently involved-hair and scalp cosmetics, nail and cuticle products, and perfumes-were similar to those reported in North American data [2]. These products are widely available in household settings and possess sensory characteristics that are attractive to young children, such as fragrances, vivid colors, and accessible packaging [2,18,19], thereby increasing the risk of accidental exposure.

Cosmetic poisoning recorded in Datatox increased in Brazil between 2019 and 2025 and predominantly affected young children through accidental oral exposures. The epidemiological pattern was characterized by a predominance of mild cases and a low frequency of severe or fatal outcomes. These findings contribute to the epidemiological characterization of cosmetic poisoning in Brazil and may support toxicological surveillance, cosmetovigilance, and prevention efforts targeting the most exposed populations.

## Data Availability

The aggregated database used in this study was deposited in the SciELO Data repository and is available at: https://doi.org/10.48331/SCIELODATA.ZT6PK4. The data correspond to the extraction performed from the public Datatox/Abracit dashboard on 1 January 2026, at 07:23.
